# Why Is Long-Term Therapy Required to Cure Tuberculosis?

**DOI:** 10.1371/journal.pmed.0040120

**Published:** 2007-03-20

**Authors:** Lynn E Connolly, Paul H Edelstein, Lalita Ramakrishnan

## Abstract

The authors argue that understanding and countering general bacterial mechanisms of phenotypic antibiotic resistance may hold the key to reducing the duration of treatment of all recalcitrant bacterial infections, including tuberculosis.

A fundamental problem in the treatment of tuberculosis (TB) is the long duration of therapy required for cure. The recalcitrance of Mycobacterium tuberculosis (MTB) to eradication is thought to result from its achieving a nonreplicating (dormant) state in the host. Because virtually all classes of antibiotics require bacterial replication for their action, the nonreplicating state is thought to render MTB phenotypically resistant to otherwise bactericidal antibiotics.

Tuberculosis drug discovery efforts have been guided by the belief that MTB achieves this nonreplicating state as the result of specific interactions with the host, particularly residence in certain types of tuberculous granulomas. This belief has placed the imperative on understanding and overcoming TB-specific mechanisms by which the nonreplicating state is achieved. Yet, it is also known that many other pathogenic bacteria display phenotypic drug resistance in vivo, accounting for the need for longer durations of antibiotic therapy than would be predicted from the time required for in vitro bacterial killing. Only recently has attention been given to these general mechanisms to explain the need for prolonged TB therapy.

In this article, we consider general versus MTB-specific models of phenotypic antibiotic resistance (see Glossary) in light of our review of human TB treatment data. These data suggest that the duration of therapy required for cure correlates with overall bacterial burden. This correlation between bacterial burden and time to cure is not unique to TB, as it has been found in other bacterial infections, both acute and chronic. High bacterial burden infections, in turn, are associated with an increased frequency of phenotypically drug-resistant bacteria. We argue that understanding and countering general bacterial mechanisms of phenotypic antibiotic resistance may hold the key to reducing the duration of treatment of all recalcitrant bacterial infections, including TB.

## Types and Consequences of MTB Drug Resistance

Soon after the discovery of streptomycin it became clear that while many patients with TB treated with this drug initially improved dramatically, most developed streptomycin-resistant strains so that there was little improvement in mortality over untreated patients [1]. The development of new antibiotics led to the realization that there were two requisites for effective cure: treatment with multiple antibiotics and long therapy [2]. Indeed, the minimum length of treatment and number of drugs required for cure has been more carefully tested for TB than for most infectious diseases (see [3] and Table S1).

**Glossary**

**Antibiotic indifference:** A subtype of phenotypic resistance to antibiotics due to decreased or absent bacterial growth of the entire bacterial population, generally in response to adverse environmental conditions, such as host defense reactions.

**Biofilms:** Multicellular bacterial communities encased in a matrix which demonstrate resistance to antibiotic killing in the absence of genetic resistance mechanisms.

**Dormancy:** A nonreplicating state, thought to be achieved by M. tuberculosis in the host, that renders the bacteria phenotypically resistant to killing by both host immune mechanisms and antibiotics.

**Latency:** Clinically asymptomatic infection with M. tuberculosis.

**Persisters:** A stochastically determined subset of bacteria that arise in an otherwise growing population of bacteria and are in a state of slow or non-growth, rendering them resistant to antibiotics.

**Phenotypic antibiotic resistance:** A general term for the phenomenon by which genetically homogeneous, antibiotic-susceptible bacterial populations (or subpopulations) become transiently insensitive to antibiotic killing.

The need for multidrug and long-term therapy stems from two different drug resistance mechanisms. MTB can exhibit genetic resistance that is heritable and fixed, as well as phenotypic, reversible resistance to administered antibiotics. The presence of genetic drug resistance in some or all of the infecting bacteria dictates the need for multidrug therapy [2,4]. The greater the bacterial burden, the more likely that it contains genetically resistant mutants [5]. Therapy failure due to genetic resistance is related to the frequency of preexisting resistant mutants and their enrichment by selective pressures imposed by inadequate therapy [4]. Simultaneous use of multiple anti-TB drugs makes it less likely that a mutant resistant to a single agent will survive.

MTB also exhibits phenotypic drug resistance. In patients who relapse early after appropriate multidrug therapy, the bacteria remain genetically susceptible to the initial antibiotics and cure is achieved by additional treatment with the same regimen [6,7]. This phenomenon may be due to a subpopulation of nonreplicating bacteria that survives until antituberculous therapy is stopped and causes relapse as it resumes growth in the absence of antibiotics. Long-term antibiotic treatment may cure the infection by eradicating these bacterial populations as they periodically leave the nonreplicating state. Further supporting this theory of MTB's development of a nonreplicating and therefore phenotypically resistant state in vivo is the observed discrepancy between in vitro and in vivo antibiotic killing [8].

Treatment of MTB with isoniazid (INH), a drug that targets cell wall synthesis, causes a 3-log reduction in broth culture in two hours [9,10], whereas more than 14 days of therapy are required to achieve a 3-log reduction in viable bacterial counts in the sputum during active TB [11,12] and several months of treatment are required to eradicate latent TB (Table 1). The role of pyrazinamide in shortening TB therapy to six months may also suggest the existence of a nonreplicating population in vivo, as, unlike other anti-TB drugs, pyrazinamide is more active against nonreplicating than actively replicating MTB in vitro [13,14]. Phenotypic antibiotic resistance likely accounts for the need for longer antibiotic therapy in many bacterial infections, presenting a universal obstacle to the treatment of infectious diseases ([15–17] and Table 1).

**Table 1:**
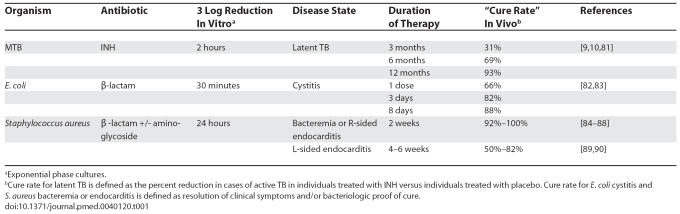
Phenotypic Antibiotic Resistance In Vivo Is Common to Many Bacterial Pathogens

The impact of phenotypic drug resistance on TB treatment outcomes is particularly dire. In the absence of an effective vaccine, TB eradication is dependent on curing infected individuals who are either contagious or may become contagious after reactivation of latent infection. The relative lack of protective immunity provided by natural infection makes control all the more dependent on complete bacterial eradication from the population, since individuals who are cured of TB remain vulnerable to reinfection [18,19]. Drug resistance, and the consequent need for long-term multidrug therapy have stymied TB eradication efforts particularly in poor countries with the highest disease burden. Poor adherence to therapy also has led to an alarming increase in multidrug-resistant (MDR) and extensively drug-resistant (XDR) strains [20], which are associated with high morbidity and mortality [21,22]. Hence the critical need for new drugs to shorten treatment of drug-sensitive TB, and to treat MDR- and XDR-TB.

## Overview of TB Pathogenesis and Pathology

A review of TB pathogenesis and pathology will facilitate the assessment of the models proposed for the mechanisms of phenotypic antibiotic resistance of MTB. MTB reaches the alveoli in small, aerosolized particles and is transported into tissues within host macrophages, which aggregate with other immune cells to form granulomas, the hallmark lesion of TB. In immunocompetent individuals, there are two main outcomes of initial infection: the development of active TB or the establishment of a clinically asymptomatic (latent) infection. Active disease is associated with a wide range of granuloma structures [23–25], including bacteria-laden, necrotic (caseating) lesions undergoing central liquefaction and large open cavities. Patients with active disease also harbor lesions in various stages of healing, including closed granulomas with hard, central caseum, and fibrotic and calcified lesions. These latter types of lesions with lower bacterial burdens [23,26] are the only lesion types detected in latent TB [23]. However, the actual physical location of viable bacteria during latent infection remains a topic of considerable debate. In latently infected individuals, viable bacteria or bacterial DNA have been detected outside of granulomas in apparently normal tissue [26,27]. In contrast, immunocompromised (e.g., HIV-infected) individuals tend to develop disease with poorly organized, noncaseating lesions that contain numerous bacteria [28].

In summary, during active disease, numerous bacteria are found in highly organized, caseating, and cavitary lesions of immunocompetent individuals or in poorly organized, noncaseating granulomas of severely immunocompromised individuals, whereas the lesions present in latent TB contain few bacteria and viable bacteria may be present outside of discernible granulomas. The lesions characteristic of latency are also found in immunocompetent individuals with active disease.

## TB-Specific Model of In Vivo–Induced Phenotypic Antibiotic Resistance

The first step in understanding MTB phenotypic drug resistance is to address whether it is mediated by TB-specific mechanisms as has been widely postulated, or by mechanisms common to all bacteria. TB-specific models suggest that environmental conditions in specific granuloma types, in particular those associated with latent disease, induce nonreplicating bacterial populations and thereby antimicrobial resistance [29–33]. This nonreplicating state is thought to be an MTB-specific response to conditions found within closed granulomas such as hypoxia and/or nitric oxide production. According to this model, exposure to these conditions leads to the expression of a discrete set of genes known as the dormancy regulon that are in turn responsible for maintaining the bacilli in the nonreplicating and hence resistant state [29,34–36]. The theory that TB-specific, environmentally induced mechanisms lead to sustained phenotypically drug-resistant bacterial populations has led to an emphasis on understanding specific host environments such as hypoxia and the specific bacterial gene expression programs they induce as a basis for developing drugs that intercept this host–bacterial interface [29–33,35–37].

The main argument favoring the environmentally induced in vivo dormancy program specific to MTB is based on observed differences in bacterial growth in vitro depending on the type of human lesion from which the bacteria were isolated [24,25]. Cavitary lesions resected from treated patients contained drug-resistant MTB that grew in a normal time frame (eight weeks), whereas “latent”-appearing (closed caseous) lesions from these same patients often contained drug-sensitive bacteria that grew only after three to ten months of incubation. These observations were interpreted to mean that the bacteria from the cavitary lesions were actively replicating and thus susceptible to the administered antibiotics. Therefore, following antibiotic therapy, this niche became populated by the growth of the drug-resistant mutants that were selected for during drug treatment.

In contrast, the bacteria in the closed lesions were thought to have been driven into a nonreplicating state by adverse conditions present within the lesion prior to antibiotic therapy. The nonreplicating state of the bacteria in these lesions was felt to account for both characteristics observed in vitro: the slower growth due to the need to overcome this dormant state as well as drug sensitivity owing to their never having been acted upon by the antibiotic in vivo. These differences were felt to be unlikely to be due to a lack of drug exposure in the closed lesions, because several of the agents used have been shown to penetrate both types of lesions [23,38]. However, these findings could have had an alternate explanation, which was not considered. The slower growing, drug-sensitive bacteria could have been present in the open lesions but their in vitro detection masked by the more rapidly growing, drug-resistant bacteria. If true, this would mean that the nonreplicating state is not specifically induced by the environment present in the closed lesions but is present in all bacterial populations and lesion types in vivo.

## Do Human Pulmonary TB Treatment Trials Inform Us about Mechanisms of Phenotypic Antibiotic Resistance?

**Figure 1:**
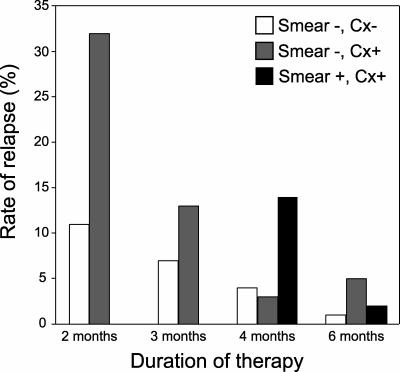
Duration of Curative Therapy for Pulmonary Tuberculosis Correlates Directly with Organism Burden Pulmonary TB with a low bacterial burden (smear negative, culture negative [Cx-]; white bars) requires the shortest duration of four-drug therapy to achieve relapse rates <10%. Moderate burden (smear negative, culture positive [Cx+]; grey bars) patients require intermediate treatment courses, while high burden (smear positive, Cx+; black bars) cases require the longest duration of therapy. All patients were HIV negative and were treated with six months of therapy consisting of streptomycin (S), isoniazid (H), rifampin (R), and pyrazinamide (Z) during the intensive phase, followed by SHRZ or HR combinations in the continuation phase. Data for the figure were obtained from references [78–80,91–95].

**Figure 2:**
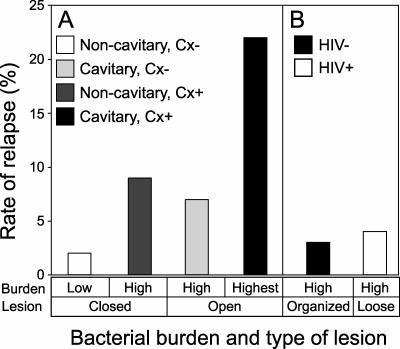
Duration of Curative Therapy Is Longest for Disease States Associated with High-Organism Burden Lesions (A) Active TB associated with cavitary lesions requires longest duration of therapy to cure. Treated pulmonary TB with open lesions and the highest bacterial burden (cavitary lesions with positive sputum cultures [Cx+] after two months of therapy; black bars) is associated with higher rates of relapse than are disease states associated with closed lesions (noncavitary, culture negative [Cx-] at two months; white bars, and noncavitary, Cx+ at two months; dark gray bars) or with lower bacterial burdens (noncavitary, Cx- at two months; white bars, or cavitary, Cx- at two months; light gray bars). All patients were HIV negative and received isoniazid (H), rifampin (R), pyrazinamide (Z), and ethambutol (E) or streptomycin (S) during the intensive phase followed by HR or H plus rifapentine (P) in the continuation phase. (B) Active disease states characterized by well-organized (HIV-) or poorly organized (HIV+) lesions require the same duration of therapy for cure. The relapse rate for high-burden (smear-positive) disease states associated with well-organized, cavitary lesions (HIV-; black bars) or loosely organized, noncaseating lesions (HIV+; white bars) treated with standard therapy (EHRZ for two months followed by HR for four months) is the same. Data for HIV+ and HIV- individuals were obtained from references [96,97].

Another problem with the TB-specific model is that it implicates the lesions that are associated with few bacteria in inducing bacterial phenotypic antibiotic resistance. However, short-course treatment trials for pulmonary TB suggest that the duration of treatment required to prevent relapse of active disease is directly proportional to the organism burden, rather than the predominant type of granuloma microenvironment present (Figures 1 and 2, Table S1). Smear-positive and cavitary disease states are associated with the highest organism burden [12,39] and require the longest duration of therapy to effect cure (Figures 1 and 2). In contrast, both HIV-positive and HIV-negative individuals with latent TB, which is characterized by low bacterial burden, are readily “cured” with single-drug therapy (Table S1). Twelve months of INH therapy in adherent populations with a low risk of reinfection leads to a 92%–93% reduction in the rate of active disease [40,41]. This reduction, using a single drug, is comparable to that seen in treating high-burden, active disease with multidrug therapy, underscoring the importance of bacterial burden as one of the main determinants of successful treatment. The high relapse rate of cavitary disease may also be related to poor penetration of the cavity by antibiotics, due to the dense, fibrous capsule surrounding these lesions [23]. However, some studies have shown that antibiotics do penetrate such lesions [23,38]. This point is underscored by the growth of resistant bacteria from these lesions in the studies described in the previous section [24,25].

An alternative explanation for the long duration of therapy required to treat active disease states associated with the highest bacterial burden (e.g., smear positive and cavitary disease) is that those individuals with active disease have a greater burden of bacteria present in “latent-type” lesions than do those with low burden or latent disease. According to this interpretation, it is the total burden of bacteria present within these “latent” lesions that dictates the duration of therapy rather than the overall burden. However, this interpretation fails to explain the finding that HIV-infected individuals who tend to have poorly formed, noncaseating granulomas with high bacillary burdens [28] also require long durations of therapy (Figure 2 and Table S1) [28]. Further, the lesion-specific model does not account for the findings that MTB exhibits nonreplicating states [42,43] and phenotypic drug resistance [44] during experimental infection of the mouse, an animal that forms poorly organized macrophage and lymphocyte aggregates that do not resemble human, caseating lesions. Together, these observations are most consistent with the conclusion that phenotypically drug-resistant MTB is present in all lesions rather than being restricted to “latent” granuloma environments.

## Non-TB Specific Models to Explain the Problem of Phenotypic Drug Resistance

The human treatment trial data are most readily explained by a model in which infections characterized by the highest organism burden (be it in cavitary lesions, caseating lesions undergoing liquefaction, or poorly formed noncaseating granulomas typical of advanced HIV coinfection) also have the highest number of phenotypically drug-resistant bacteria. Because high organism burden is associated with phenotypic resistance in other infectious diseases, we propose that the mechanisms are similar in MTB and other pathogenic bacteria. We will describe the possible mechanisms briefly here; for more detailed reviews of specific mechanisms see references [15–17,45,46].

Killing rates of actively growing MTB cultures are dramatically greater than killing rates of stationary-phase MTB cultures, in which the bacteria are resistant to killing in the absence of genetic resistance mechanisms [9,10]. This phenomenon had previously been described in other bacterial systems and the term antibiotic indifference was coined to describe the finding that bacteria that are not dividing, due to some inhibitory environmental condition, are resistant to killing by most antibiotics [47]. This phenomenon is not limited to in vitro culture systems. For example, the dose of penicillin required to cure experimental infections in animals is proportional to the total bacterial burden (both inoculum size and duration of infection). As the infection progresses, bacterial growth slows and eventually stops due to a variety of inhibitory conditions encountered in the host, rendering the residual population phenotypically antibiotic resistant [47–49]. One potential mechanism for MTB dormancy and phenotypic antibiotic tolerance in vivo is the development of antibiotic indifference in response to host defense mechanisms or nutrient deprivation. Although nutrient deprivation has long been proposed as one of the signals leading to mycobacterial dormancy, this mechanism need not be restricted to specific granuloma pathologies and is clearly not unique to MTB.

In addition to drug indifference, non-inherited drug resistance can also be explained by the observation that populations of actively growing bacteria contain a specialized, nonreplicating subpopulation known as persister cells [50]. Like drug-indifferent bacteria, these persister cells remain genetically drug sensitive but are phenotypically drug resistant, and their number increases with total organism burden [51]. Although the initial establishment of the persister population is likely to be stochastic [52], the magnitude of this population may be further influenced by specific conditions operant in vivo such as growth in macrophages [53] or biofilms [54]. For example, Legionella pneumophila grown in macrophages is more antibiotic resistant than broth-grown bacteria, suggesting that intramacrophage growth enriches phenotypically antibiotic-resistant populations [53]. This might occur because host-killing mechanisms may also target actively replicating bacteria [46]. Further, bacteria that have incurred DNA damage, perhaps as a result of host-killing mechanisms, undergo replication arrest to allow for DNA repair, rendering them transiently insensitive to antibiotic killing [55]. Some antibiotics actually induce DNA repair systems [56], halting bacterial division and theoretically rendering these bacteria even more resistant to therapy.

The molecular mechanisms of persister formation are beginning to be elucidated and include growth arrest secondary to the action of toxin–antitoxin modules [57,58]. The toxin portion of these modules acts to cleave mRNAs positioned in the ribosome, leading to translational and growth arrest [59]. Numerous putative toxin–antitoxin modules are found in the MTB genome [60,61] and some of these have been shown to cause translational arrest when expressed in Escherichia coli [62], but their role(s) in persister formation, in vivo survival, and/or drug indifference are unknown. *relA*, a gene involved in the bacterial response to starvation, has also been shown to play a role in some types of persister formation in E. coli [63]. Although a potential role for *relA* in mycobacterial phenotypic drug resistance has not been shown, *relA* is required for chronic infection in the mouse model of TB [64], suggesting a possible connection to mechanisms of resistance to both host- and antibiotic-mediated killing.

Studies of other chronic bacterial infections suggest that biofilm formation is responsible for the relative in vivo resistance to antibiotic killing [65]. Biofilms are multicellular bacterial communities encased in a matrix and bacteria within biofilms are phenotypically resistant to antibiotic killing when compared to growing planktonic cells [66]. Examples of important biofilm infections in humans include Pseudomonas aeruginosa lung infections in cystic fibrosis, endocarditis, and device-related infections. Key aspects of MTB biology are reminiscent of biofilm behavior. For example, MTB in liquid culture grows as large clumps of cells known as cords. The ability to cord in culture correlates with virulence [67–69], suggesting that the capacity to grow in a multicellular community is an important determinant of MTB survival in the host. MTB may also be found in a biofilm-like state in vivo. For example, large clumps of bacteria reside in an acellular matrix in certain human lesions, such as caseating lesions undergoing liquefaction [23]. Biofilms of other mycobacterial species have been shown to be phenotypically antibiotic resistant [70,71] and M. avium biofilm-associated genes are required for mouse infection [72], further supporting the notion that biofilms may play key roles in mycobacterial drug resistance and virulence.

**Five Key Papers in the Field**

1. **Canetti G, 1955** [23] A seminal text describing the pathology of human pulmonary tuberculosis with particular focus on the bacillary content of different types of lesions.
2. **Vandiviere HM et al., 1956** [25] A detailed description of growth rates and antibiotic resistance patterns of M. tuberculosis isolated from different types of human tuberculous lesions, which has strongly influenced thinking in the field regarding mechanisms of bacterial phenotypic antibiotic resistance in vivo.
3. **Balaban NQ et al., 2004** [52] Provides the first direct evidence that persisters are a preexisting population of non-dividing cells within a growing culture using ground-breaking techniques to study persisters at the single cell level.
4. **Keren I et al., 2004** [57] First paper to describe global gene expression profiles in an isolated population of antibiotic-treated persister cells, supporting the role of toxin–antitoxin modules in persister biology. Later studies of naïve persisters have confirmed these results.
5. **Hong Kong Chest Service/ Tuberculosis Research Centre, Madras/British Medical Research Council, 1984, 1987, and 1989** [78–80] A series of controlled trials of varying treatment durations for both smear-negative and -positive pulmonary tuberculosis that confirm bacillary burden to be one of the main determinants of duration of therapy required for cure.

The phenotypic resistance to antibiotics exhibited by bacteria within biofilms is likely multifactorial. Proposed mechanisms of resistance include poor antibiotic penetration of the biofilm, expression of biofilm genes that confer resistance, and the presence of different microenvironments in the biofilm that lead to different growth rates and thus differing antibiotic sensitivity [45,65]. One prominent school of thought is that the biofilm environment may also enrich the formation of persister cells [54,73]

## Summary and Proposed Areas of Research

New drugs that target nonreplicating bacteria are likely to revolutionize TB therapy. Such agents have the potential not only to treat MDR and XDR strains but also to dramatically shorten the duration of curative therapy. Shorter treatment times will likely translate into higher patient adherence, reduced transmission, and decreased drug resistance, leading in turn to diminished mortality and substantial gains in tuberculosis control efforts. For example, mathematical models based on the current situation in Southeast Asia estimate that a two month regimen could prevent ~25% of deaths and ~20% of new cases over a 18-year period compared to current treatment regimens [74].

A major obstacle for such truly short-course therapy is the development of phenotypic antibiotic resistance in vivo. This phenomenon is common to all bacteria and these resistant populations may be enriched under a variety of conditions that are operant in vivo, such as intracellular growth, DNA damage, exposure to other antimicrobials, and biofilm formation. In addition to studying possible TB-specific mechanisms of phenotypic drug resistance, we suggest that the study of mycobacterial persister formation and biofilm-like growth states may lead to drug discovery. Additionally, a better understanding of mechanisms of phenotypic antibiotic resistance in other pathogenic bacteria will likely have implications for MTB therapy. Given our current understanding, the development of antibiotics that are effective against non-dividing bacteria is of potential great importance [75]. Promising candidates under investigation include the ATP synthase inhibitor R207910 [76]; however, MTB mutants resistant to this agent have been found even before clinical trials have been completed [77]. Development of additional drugs that target bacterial or host programs that induce phenotypic antibiotic resistance mechanisms will be aided by a better understanding of the physiology of this MTB population and the conditions that induce or enrich it.

## Supporting Information

Table S1 Summary of treatment trials demonstrating direct relationship between minimal duration of curative therapy and bacterial burden (115 KB DOC).

## Abbreviations

INH: isoniazid
MDR-TB: multidrug-resistant tuberculosis
MTB: Mycobacterium tuberculosis
TB: tuberculosis
XDR-TB: extensively drug-resistant tuberculosis
