# Supplementary material for: Why Is Long-Term Therapy Required to Cure Tuberculosis?
Source: PLoS Med. 2007 Mar 20;4(3):e120. doi: 10.1371/journal.pmed.0040120 (PMC1831743; doi:10.1371/journal.pmed.0040120)
Supplement: Table S1 — (115 KB DOC). [file pmed.0040120.st001.doc]

Table S1. Duration of curative therapy is directly proportional to disease burden

| Type/stage of disease | Bacterial Burden | Drugs | Duration (mos) | Rate of active disease or relapse (%) | Follow up time (months)^a^ | References |
| --- | --- | --- | --- | --- | --- | --- |
| Latent | Very low | H  RZ | 6-12  2 | 0.2 – 8.2 HIV-  1.3 – 7.5 HIV+  2 - 5 HIV+ | 24 – 70  20 – 30  30 - 37 | [1]  [1]  [2,3] |
| Smear -  Cx- | Low | SHRZ  HR | 2  3  4  4 | 11  6 - 8  4  1 | 60  60  60  44 | [4]  [4,5]  [4]  [6] |
| Smear -  Cx + | Higher^b^ | SHRZ | 2  3  4  6 | 32  13  2 - 3  5 | 60  60  60  60 | [4]  [4,5]  [5]  [5] |
| Smear +  Cx+ | Highest^c^ | SHRZ/HR  SHRZ/HR  EHRZ/HR  EHRZ/HR | 4 (2/2)  6 (2/4)  6 (2/4)  6 (2/4) | 14  2 - 3  3 HIV-  4 HIV+ | > 60  24 – 60  24  24 | [7]  [8,9,10,11,12]  [13]  [13] |
| Smear +/-, Cx+  HIV-  Culture at 2 mos.:  1) Non-cavitary, Cx-  2) Cavitary, Cx-  3) Non-cavitary, Cx+  4) Cavitary, Cx+ | Low  High  High  Highest | HRZ(E/S)/HP or HR | 6 (2/4) | 2  7  9  22 | 28 | [14] |

H = isoniazid, R = rifampin, Z = pyrazinamide, S = streptomycin, E = ethambutol, P = rifapentine, Cx = culture

Trials regarding the treatment of active tuberculosis which were considered for inclusion in this table were identified by a Medline search (key words tuberculosis, treatment, with limits of English language, human, and clinical trial), in the reference section of papers identified by the Medline search and in a review article regarding studies on the treatment of tuberculosis undertaken by the British Medical Research Council Tuberculosis Units [15]. Inclusion criteria were: 1) pulmonary tuberculosis, 2) the use of short course (6 month) therapy with either SHRZ or EHRZ in the intensive phase, and 3) a clear definition of the patient population regarding smear and culture status (smear negative or positive, culture negative or positive) with separate analysis of each patient population. Data regarding the treatment of latent tuberculosis infection with INH were taken from a review article and in the case of HIV- individuals included both PPD+ and PPD- contacts of known cases while data for HIV+ individuals includes only those who were PPD+ [1]. Data regarding the treatment of cavitary disease are from a single article [14].

^a^Mean or median follow up times are given

^b^Estimated bacterial load 3.5 log_10_ CFU/ml sputum [16]

^c^Estimated bacterial load 5.27 – 6.98 log_10_ CFU/ml sputum [16]

**References**

1. Dooley KE, Sterling TR (2005) Treatment of latent tuberculosis infection: challenges and prospects. Clin Chest Med 26: 313-326, vii.

2. Gordin F, Chaisson RE, Matts JP, Miller C, de Lourdes Garcia M, et al. (2000) Rifampin and pyrazinamide vs isoniazid for prevention of tuberculosis in HIV-infected persons: an international randomized trial. Terry Beirn Community Programs for Clinical Research on AIDS, the Adult AIDS Clinical Trials Group, the Pan American Health Organization, and the Centers for Disease Control and Prevention Study Group. Jama 283: 1445-1450.

3. Halsey NA, Coberly JS, Desormeaux J, Losikoff P, Atkinson J, et al. (1998) Randomised trial of isoniazid versus rifampicin and pyrazinamide for prevention of tuberculosis in HIV-1 infection. Lancet 351: 786-792.

4. Hong Kong Chest Service/Tuberculosis Research Centre, Madras/British Medical Research Council (1984) A controlled trial of 2-month, 3-month, and 12-month regimens of chemotherapy for sputum-smear-negative pulmonary tuberculosis. Results at 60 months. Am Rev Respir Dis 130: 23-28.

5. Hong Kong Chest Service/Tuberculosis Research Centre, Madras/British Medical Research Council (1989) A controlled trial of 3-month, 4-month, and 6-month regimens of chemotherapy for sputum-smear-negative pulmonary tuberculosis. Results at 5 years. Am Rev Respir Dis 139: 871-876.

6. Dutt AK, Moers D, Stead WW (1989) Smear- and culture-negative pulmonary tuberculosis: four-month short-course chemotherapy. Am Rev Respir Dis 139: 867-870.

7. Singapore Tuberculosis Service/British Medical Research Council (1986) Long-term follow-up of a clinical trial of six-month and four-month regimens of chemotherapy in the treatment of pulmonary tuberculosis. Am Rev Respir Dis 133: 779-783.

8. Singapore Tuberculosis Service/British Medical Research Council (1988) Five-year follow-up of a clinical trial of three 6-month regimens of chemotherapy given intermittently in the continuation phase in the treatment of pulmonary tuberculosis. Am Rev Respir Dis 137: 1147-1150.

9. Hong Kong Chest Service/Tuberculosis Research Centre, Madras/British Medical Research Council (1987) Five-year follow-up of a controlled trial of five 6-month regimens of chemotherapy for pulmonary tuberculosis. Am Rev Respir Dis 136: 1339-1342.

10. Hong Kong Chest Service/Tuberculosis Research Centre, Madras/British Medical Research Council (1991) Controlled trial of 2, 4, and 6 months of pyrazinamide in 6-month, three-times-weekly regimens for smear-positive pulmonary tuberculosis, including an assessment of a combined preparation of isoniazid, rifampin, and pyrazinamide. Results at 30 months. Am Rev Respir Dis 143: 700-706.

11. Snider DE, Graczyk J, Bek E, Rogowski J (1984) Supervised six-months treatment of newly diagnosed pulmonary tuberculosis using isoniazid, rifampin, and pyrazinamide with and without streptomycin. Am Rev Respir Dis 130: 1091-1094.

12. East and Central African/British Medical Research Council Fifth Collaborative Study (1986) Controlled clinical trial of 4 short-course regimens of chemotherapy (three 6-month and one 8-month) for pulmonary tuberculosis: final report. Tubercle 67: 5-15.

13. Perriëns JH, St Louis ME, Mukadi YB, Brown C, Prignot J, et al. (1995) Pulmonary tuberculosis in HIV-infected patients in Zaire. A controlled trial of treatment for either 6 or 12 months. N Engl J Med 332: 779-784.

14. Benator D, Bhattacharya M, Bozeman L, Burman W, Cantazaro A, et al. (2002) Rifapentine and isoniazid once a week versus rifampicin and isoniazid twice a week for treatment of drug-susceptible pulmonary tuberculosis in HIV-negative patients: a randomised clinical trial. Lancet 360: 528-534.

15. Fox W, Ellard GA, Mitchison DA (1999) Studies on the treatment of tuberculosis undertaken by the British Medical Research Council tuberculosis units, 1946-1986, with relevant subsequent publications. Int J Tuberc Lung Dis 3: S231-279.

16. Allen BW, Mitchison DA (1992) Counts of viable tubercle bacilli in sputum related to smear and culture gradings. Med Lab Sci 49: 94-98.
